# Cytochrome *c*-peroxidase modulates ROS homeostasis to regulate the sexual mating of *Sporisorium scitamineum*

**DOI:** 10.1128/spectrum.02057-23

**Published:** 2023-10-11

**Authors:** Enping Cai, Huan Jia, Ruqing Feng, Wenqiang Zheng, Lei Li, Li Zhang, Zide Jiang, Changqing Chang

**Affiliations:** 1 Guangdong Laboratory for Lingnan Modern Agriculture, Guangdong Provincial Key Laboratory of Microbial Signals and Disease Control, South China Agricultural University, Guangzhou, Guangdong, China; 2 Integrate Microbiology Research Center, College of Plant Protection, South China Agricultural University, Guangzhou, China; Broad Institute, Cambridge, Massachusetts, USA

**Keywords:** *Sporisorium scitamineum*, cytochrome *c*-peroxidase, reactive oxygen species, pheromone response factor, mating/filamentation

## Abstract

Sugarcane smut, caused by the basidiomycetous fungus *Sporisorium scitamineum,* is a global fungal disease resulting in substantial economic losses. Our previous research has highlighted the significant role of reactive oxide species (ROS) in the sexual reproduction of *S. scitamineum*, yet the underlying mechanisms remain unclear. In this study, we identified the cytochrome *c*-peroxidase encoding gene, Ss*CCP1*, as crucial for oxidative stress resistance. We further found that absence of SsCcp1 leads to defects in mating/filamentation and disrupts intracellular ROS homeostasis. However, restoration of mating/filamentation of *SsCCP1* deletion mutants was partially achieved with exogenous antioxidants (vitamin C or vitamin E). Notably, transcription of the pheromone response factor *SsPRF1*, pivotal for mating/filamentation of *S. scitamineum*, is significantly reduced in the Ss*CCP1* deletion mutants or under exogenous hydrogen peroxide (H_2_O_2_) conditions. Nevertheless, the addition of exogenous vitamin C for 60 min significantly up-regulated the *SsPRF1* gene in the Ss*CCP1* deletion mutants. Moreover, the constitutive expression of *SsPRF1* essentially reinstated the mating/filamentation of Ss*CCP1* deletion mutants, suggesting that SsCcp1 mediates the transcriptional activity of *SsPRF1* by maintaining ROS homeostasis to regulate the mating/filamentation of *S. scitamineum*. Further investigations revealed that SsCcp1 is necessary for ROS detoxification and full pathogenicity *in planta*. Overall, this study not only unveils the importance of SsCcp1 in *S. scitamineum* mating/filamentation, ROS detoxification, and virulence but also provides insights into the role of SsCcp1-regulated ROS homeostasis in sexual reproduction of *S. scitamineum*.

**IMPORTANCE:**

Reactive oxygen species play an important role in pathogen-plant interactions. In fungi, cytochrome *c*-peroxidase maintains intracellular ROS homeostasis by utilizing H_2_O_2_ as an electron acceptor to oxidize ferrocytochrome *c*, thereby contributing to disease pathogenesis. In this study, our investigation reveals that the cytochrome *c*-peroxidase encoding gene, *SsCCP1*, not only plays a key role in resisting H_2_O_2_ toxicity but is also essential for the mating/filamentation and pathogenicity of *S. scitamineum*. We further uncover that SsCcp1 mediates the expression of SsPrf1 by maintaining intracellular ROS homeostasis to regulate *S. scitamineum* mating/filamentation. Our findings provide novel insights into how cytochrome *c*-peroxidase regulates sexual reproduction in phytopathogenic fungi, presenting a theoretical foundation for designing new disease control strategies.

## INTRODUCTION

Sugarcane smut, a worldwide fungal ailment, incurs substantial losses in cane yields and the sugar industry. Severe instances can lead to up to 30% crop failure, potentially wiping out certain varieties (1, 2). *Sporisorium scitamineum*, a basidiomycetous fungus, is the pathogen responsible for sugarcane smut disease. *S. scitamineum* has a bipolar mating system and three life cycles (comprising yeast-like haploid basidiospore, dikaryotic hypha, and diploid teliospore). Mating of haploids of two opposite mating types, *MAT-1* and *MAT-2*, leads to the formation of a dikaryotic hypha that infects and colonizes the host sugarcane, ultimately yielding diploid teliospore through nuclear fusion (3, 4).

What is crucial to the pathogenicity of *S. scitamineum* is interlinked with sexual reproduction, as only the resulting dikaryotic hyphae after sexual mating can invade the host sugarcane (4, 5). This underscores the importance of sexual mating in the pathogenicity of *S. scitamineum*. The fungus *S. scitamineum* is similar to *Ustilago maydis* in that the mating/filamentation is regulated by genes responsible for the *a* and *b* mating type genes (6, 7). The *a* locus genes encode lipopeptic pheromone precursor Mfa and receptor Pra forming an intercellular pheromone-receptor recognition system (8, 9). This system is necessary for haploid sporidia recognition and fusion, while the multiallelic *b* locus encodes heterodimeric transcription factor subunits (bE and bW) that regulate filamentation growth (8, 10). The pheromone responsive factor Prf1 regulates the *a* and *b* mating type genes, which binds to the pheromone response elements present in promoter regions of these genes (11). The activity of the pheromone responsive factor Prf1 is transcriptionally regulated by mitogen-activated protein kinase (MAPK) and protein kinase A (PKA) module (12 – 14).

In organisms, ROS are produced as byproducts of metabolic processes or under adverse conditions, and they are necessary for defense reactions in pathogen-host interactions (15 – 17). Pathogens have evolved sophisticated ROS-producing and scavenging systems to facilitate host infection (18, 19). Peroxidase is a class of enzymes that catalyze the reduction of H_2_O_2_ to water by oxidizing various substrate molecules, playing an important role in maintaining the redox balance of cells and participating in oxidative stress response (20). Cytochrome *c*-peroxidase 1 (Ccp1) is the first heme peroxidase whose crystallographic structure was determined (21, 22). The yeast protein cytochrome *c*-peroxidase precursor is hydrolyzed by the mAAA protease subunit Yta10, Yta12, and Pcp1, which removes its N-terminal presequence yielding the mature Ccp1 protein (23, 24). In anoxic *Escherichia coli*, Ccp1 is essential for the degradation of H_2_O_2_, allowing it to grow on a nonfermentable carbon source when supplied with H_2_O_2_ (25). *Citrobacter rodentium* utilizes host NoX1-derived H_2_O_2_ to promote pathogen growth through Ccp1-mediated anaerobic respiration (26). Cytochrome *c*-peroxidase in *Saccharomyces cerevisiae* is primarily used as a mitochondrial H_2_O_2_ sensing and signaling protein to maintain ROS homeostasis (22, 27, 28). In pathogenic fungi, Ccp1 of *Candida albicans* is a negative regulator of hyphal growth and regulates intracellular ROS and methylglyoxal levels by inducing the activity of erythroascorbate peroxidase and glutathione-related enzymes (29). In *Cryptococcus neoformans*, Ccp1 contributes to the resistance to exogenous oxidative stress, but *Ccp1* deletion mutants are not less virulent to mice (30). Ccp1 in *Paraccidioides brasiliensis* is activated by H_2_O_2_ to prevent cellular oxidative damage (31). In *Botrytis cinerea*, cytochrome *c*-peroxidase was secreted to scavenge plant-produced H_2_O_2_ and promote pathogen invasion (32). However, few reports have described the function of cytochrome *c*-peroxidase in smut fungi.

In this study, we identified the gene encoding cytochrome *c*-peroxidase (*CCP1*) in *S. scitamineum* and performed functional analysis through reverse genetics and phenetics. Our findings demonstrated that SsCcp1 is required for ROS detoxification, mating/filamentation, and pathogenicity of *S. scitamineum*. Further investigations indicate that SsCcp1 mediates the *SsPRF1* expression via maintaining intracellular ROS homeostasis to regulate mating/filamentous in *S. scitamineum*. Overall, our data suggest that SsCcp1 is essential for maintaining intracellular ROS homeostasis to regulate sexual coordination and pathogenicity of *S. scitamineum*.

## RESULTS

### SsCcp1 is necessary for oxidative stress resistance in *S. scitamineum*

**Fig 1:**
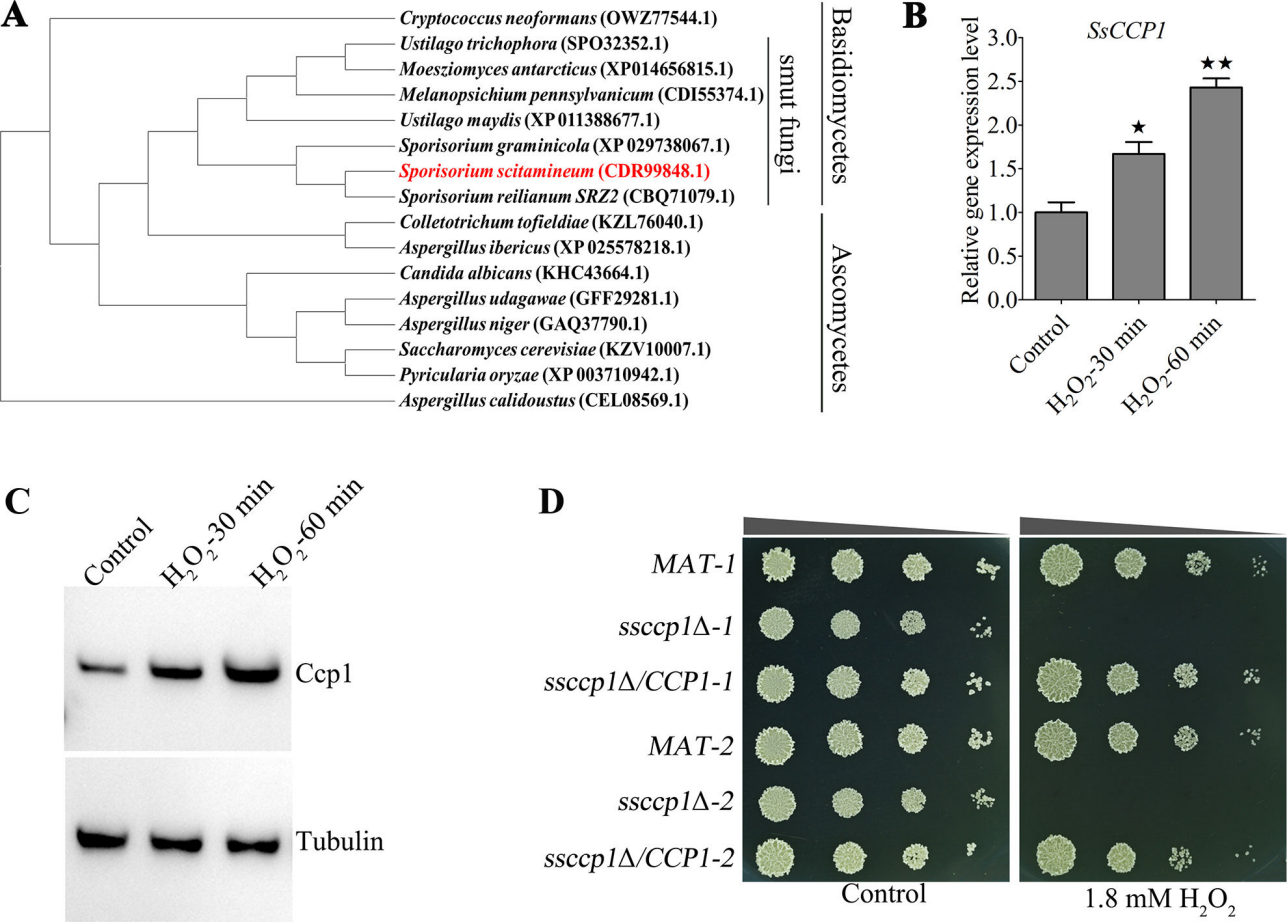
The role of SsCcp1 in oxidative stress resistance. (**A**) Amino acid sequences of SsCcp1 protein were used in a BLASTP search to identify orthologous proteins. A phylogenetic tree of Ccp1 proteins from various species, including *Melanopsichium pennsylvanicum* (CDI55374.1), *Ustilago trichophora* (SPO32352.1), *Moesziomyces antarcticus* (XP014656815.1), *Sporisorium scitamineum* (CDR99848.1), *Sporisorium reilianum SRZ2* (CBQ71079.1), *Sporisorium graminicola* (XP 029738067.1), *Ustilago maydis* (XP 011388677.1), *Aspergillus calidoustus* (CEL08569.1), *Cryptococcus neoformans* (OWZ77544.1), *Saccharomyces cerevisiae* (KZV10007.1), *Pyricularia oryzae* (XP 003710942.1), *Aspergillus udagawae* (GFF29281.1), *Aspergillus niger* (GAQ37790.1), *Candida albicans* (KHC43664.1), *Colletotrichum tofieldiae* (KZL76040.1), and *Aspergillus ibericus* (XP 025578218.1), was constructed using the maximum likelihood method using MEGA 7.0 software. (**B** and **C**) The transcriptional expression of SsCcp1 was analyzed by qRT-PCR and Western blot in the *MAT-1* strain treatment with or without H_2_O_2_. The fresh haploid sporidia of *MAT-1* were allowed to grow on liquid minimal medium treatment with H_2_O_2_ for 0, 30, and 60 min. Total RNA was extracted for qRT-PCR analysis, and total protein was extracted for Western blot analysis. The level of SsCcp1 was determined with anti-Ccp1 antibody, using anti-tubulin antibody as an internal control. Relative gene expression level was calculated using the −ΔΔCt method with the *ACTIN* gene as an internal control. Statistical significance was calculated by analysis of variance (ANOVA), followed by Tukey’s multiple-comparison test. Error bars represent the standard error of mean (SEM). Bar chart depicts the statistical difference among the mean values (^★^ *P* < 0.05, ^★★^ *P* < 0.01). Three independent biological repeats were performed with three technical duplicates each. (**D**) Assessment of tolerance toward oxidative stressful conditions for the wild-type, Ss*CCP1* deletion mutant, and Ss*CCP1* genetic complementation strains. The fresh haploid sporidia of the strains indicated on the left were cultured in yeast extraction-peptone-sucrose (YePS) medium at 28°C until OD_600_ of 1.0 and then serially diluted (1 to 10^3^) to spot onto YePS plates with or without 1.8 mM H_2_O_2_. Images were taken 3–4 days after cultivation. Three independent biological repeats with two replicates were performed, and representative images were displayed.

We previously found that a cytochrome *c*-peroxidase (sequence ID: CDR99848.1) encoding gene *SsCCP1* was up-regulated under oxidation condition in *S. scitamineum*. Subsequently, phylogenetic analysis of fungal orthologous Ccp1 proteins indicated that SsCcp1 was highly conserved with its orthologs in *Sporisorium reilianum* (CBQ71079.1) and members of the smut fungi, phylogenetic clade, while less conserved with that in *Saccharomyces cerevisiae* (KZV10007.1) and members of the Ascomycetes (Fig. 1A). These results suggest that SsCcp1 is a conserved peroxidase protein. Based on the above analysis, we examined the expression dynamics of *SsCCP1* under H_2_O_2_ treatment in the wild-type *MAT-1* strain. The results showed that the transcription levels of *SsCCP1* were remarkably induced upon H_2_O_2_ treatment (Fig. 1B). Similarly, protein levels of SsCcp1 were also significantly induced under oxidative stress condition (Fig. 1C). To further examine the role of SsCcp1, we generated Ss*CCP1* deletion mutants (*ssccp1*Δ*-1* and *ssccp1*Δ*-2*) and Ss*CCP1* genetic complementation strains (*ssccp1*Δ/*CCP1-1* and *ssccp1*Δ/*CCP1-2*) by a homologous recombination approach. These mutants were performed using PCR amplification, Southern blotting, and quantitative real-time PCR (qRT-PCR) methods to confirm the successful genetic deletion and reintegration of Ss*CCP1* ( Fig. S1A, B, and C ). Details of *S. scitamineum* wild-type, Ss*CCP1* deletion mutant, and Ss*CCP1* genetic complementation strains in this study were listed in Table S1. We measured the tolerance of *SsCCP1* deletion mutants to oxidative stress on yeast extraction-peptone-sucrose-agar (YePSA) medium in the presence of 1.8 mM H_2_O_2_. Strikingly, both *ssccp1*Δ*-1* and *ssccp1*Δ*-2* displayed severe growth inhibition under H_2_O_2_ stress conditions compared to the wild-type and Ss*CCP1* genetic complementation strains (Fig. 1D). In summary, these results suggest that SsCcp1 is essential for oxidative stress resistance in *S. scitamineum*.

### SsCcp1 acts independently of Hog1-MAPK pathway in oxidative stress resistance

**Fig 2:**
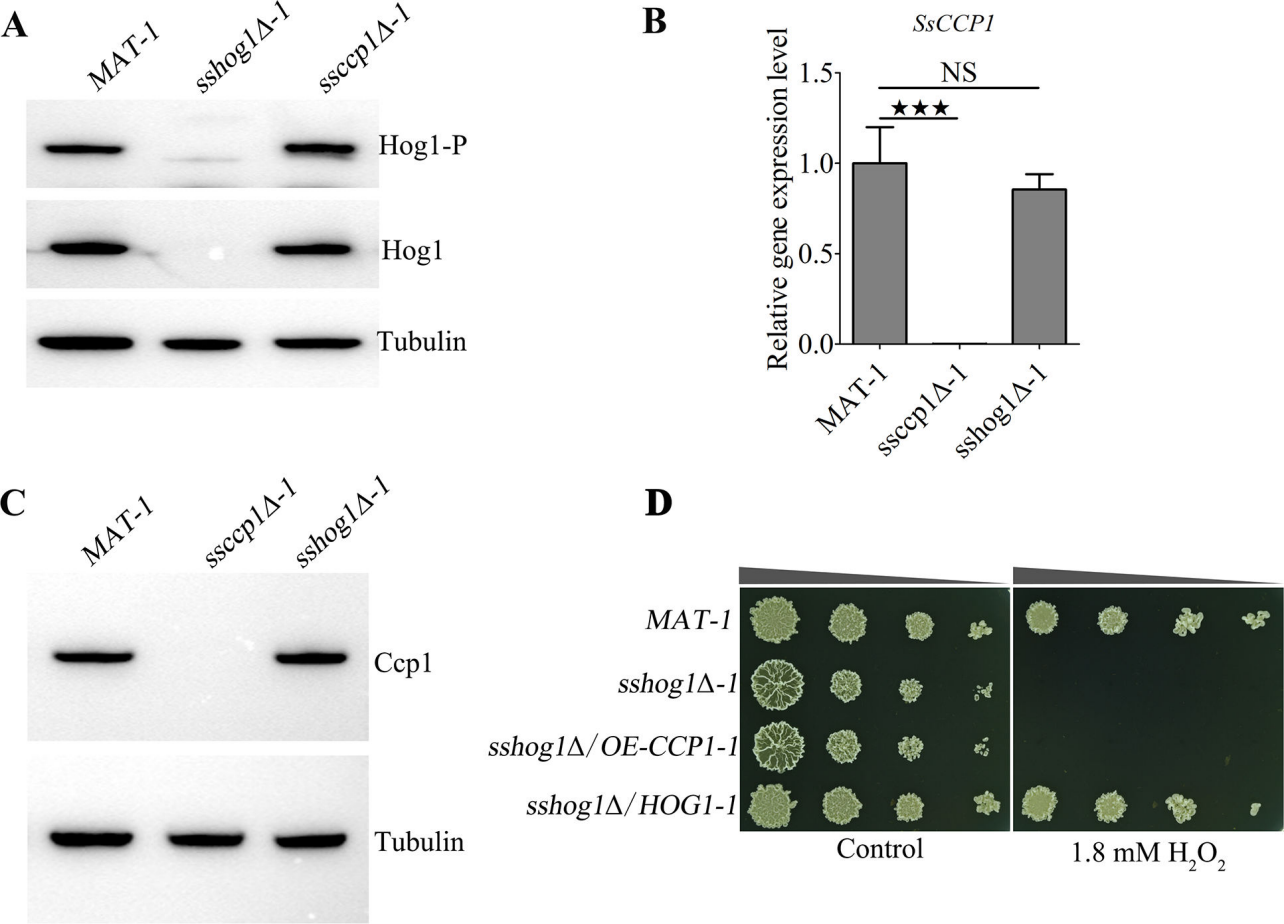
Relationship between SsCcp1 and Hog1-MAPK pathway. (**A**) The phosphorylation levels of SsHog1 in the *MAT-1*, *sshog1*Δ*-1*, and *ssccp1*Δ*-1* strains. Total protein was extracted from the fresh haploid sporidia under YePSA medium. Phosphorylated SsHog1 was detected with the primary antibody phospho-p38 MAPK (Thr180/Tyr182), and the total level of SsHog1 was determined with anti-Hog1 antibody by Western blot analysis. Three independent biological repeats were performed, and representative images were displayed. (**B**) qRT-PCR analysis of Ss*CCP1* gene in the *MAT-1*, *ssccp1*Δ*-1*, and *sshog1*Δ*-1* strains. The fresh haploid sporidia of the strains were allowed to grow on minimal medium (MM) plates for 24 h, and then, total RNA was extracted for analysis by qRT-PCR. Relative gene expression level was calculated using the −ΔΔCt method with the *ACTIN* gene as an internal control. Statistical significance was determined by ANOVA, followed by Tukey’s multiple-comparison test. Error bars represent the SEM. Bar chart depicts the statistical difference among the mean values (^★★★^ *P* < 0.001). NS denotes not statistically significant difference. Three independent biological repeats were performed with three technical duplicates each. (**C**) Western blot analysis of SsCcp1 protein levels in the *MAT-1*, *ssccp1*Δ*-1*, and *sshog1*Δ*-1* strains. The fresh haploid sporidia of the strains indicated on the above were allowed to grow on MM plates for 24 h, and then, total protein was extracted for Western blot analysis. The total level of SsCcp1 was determined with anti-Ccp1 antibody, using anti-tubulin antibody as an internal control. Three independent biological repeats were performed. (**D**) Assessment of tolerance toward oxidative stressful conditions for the *MAT-1*, *sshog1*Δ*-1*, *sshog1*Δ/*OE-CCP1-1*, and *sshog1*Δ/*HOG1-1* strains. The fresh haploid sporidia of the strains indicated on the left were cultured in YePS medium at 28°C until OD_600_ of 1.0 and then serially diluted (1 to 10^3^) to spot onto YePS plates with or without 1.8 mM H_2_O_2_. Images were taken 3–4 days after cultivation. Three independent biological repeats with two replicates were performed, and representative images were displayed.

We also studied the effect of SsCcp1 on the Hog1 MAPK pathway, which plays a crucial role in oxidative stress (33), by analyzing the phosphorylation level of SsHog1 in the *ssccp1*Δ*-1* mutant. However, the results showed that the phosphorylation level of SsHog1 in *ssccp1*Δ*-1* mutants was comparable to that of the wild type (Fig. 2A). Consistent with these observations, deletion of *SsHOG1* did not affect the transcriptional expression of *SsCCP1* (Fig. 2B and C). In addition, overexpression of *SsCCP1* gene in the *sshog1*Δ*-1* mutant failed to restore resistance to oxidative stress (Fig. 2D). Taken together, SsCcp1 is involved in oxidative stress resistance independently of Hog1-MAPK pathway in *S. scitamineum*.

### Loss of SsCcp1 results in imbalance of intracellular ROS homeostasis

**Fig 3:**
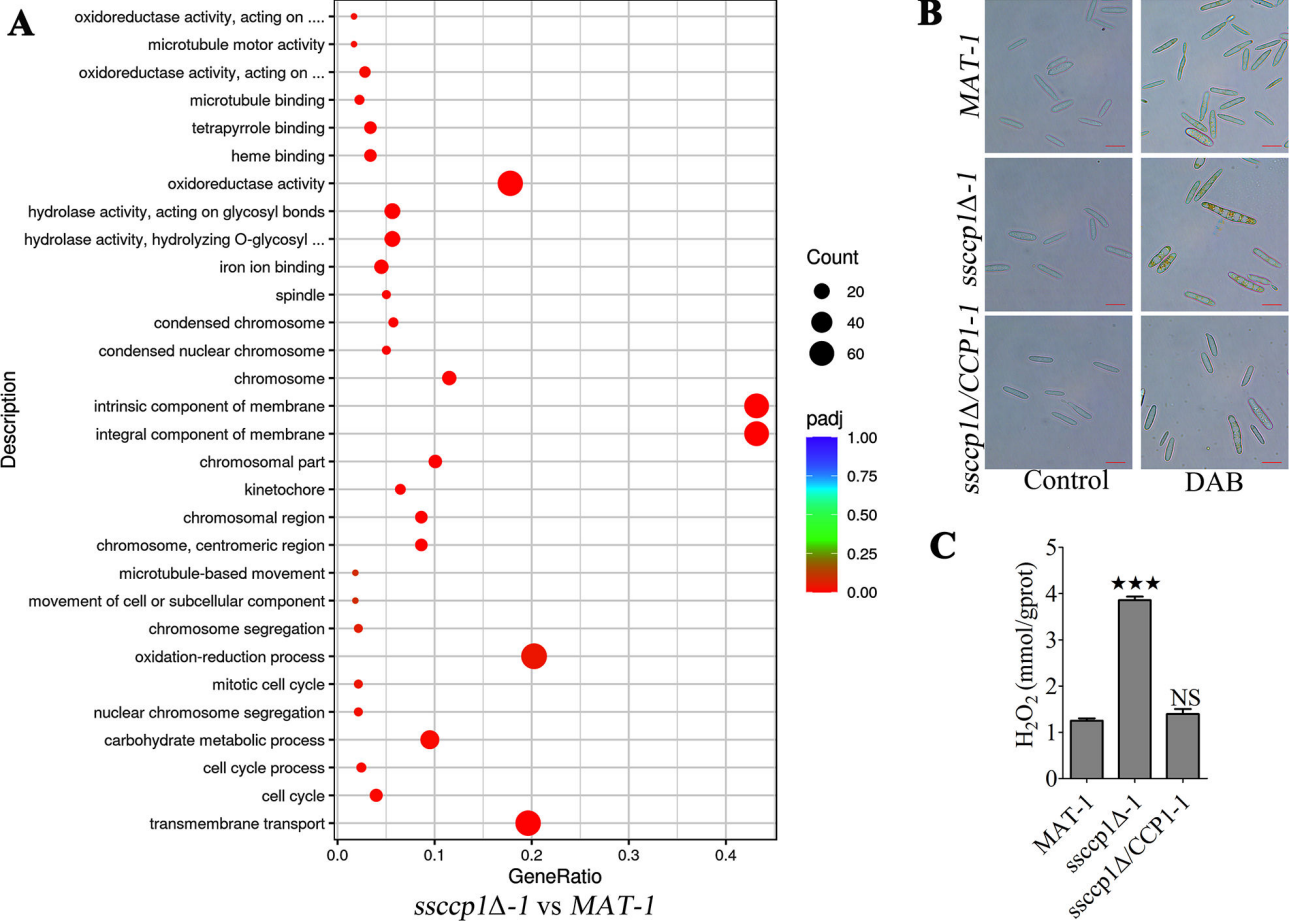
Effects of SsCcp1 on intracellular ROS homeostasis. (**A**) Gene ontology enrichment analysis of DEGs in *MAT-1* and *ssccp1*Δ*-1* strains. The bubble charts illustrate the terms in which the DEGs were enriched. A *P*-value <0.05 was considered statistically significant. (**B**) Visualization of intracellular ROS response by DAB staining. The fresh haploid sporidia of the strains indicated on the left were stained with or without DAB dye for 5 min. Images were captured at the same magnification. Scale bar is 10 µm. Three independent biological repeats with two replicates were performed, and representative images were displayed. (**C**) Measurement of intracellular H_2_O_2_ in *MAT-1*, *ssccp1*Δ*-1*, and *ssccp1*Δ/*CCP1-1* strains. The fresh haploid sporidia of the strains were allowed to grow on MM plates for 24 h and then measured H_2_O_2_ concentration. Statistical significance was determined using ANOVA, followed by Tukey’s multiple-comparison test. Error bars represent the standard deviations (SDs). Bar chart depicts the statistical difference among the mean values (^★★^ *P* < 0.01). NS denotes not statistically significant difference. Three independent experiments were performed in triplicate.

To further elucidate the effect of SsCcp1 on intracellular ROS homeostasis, we conducted a comparative transcriptome analysis using the wild-type *MAT-1* and the *ssccp1*Δ*-1* mutant sporidia grown in YePSA medium. Differentially expressed genes (DEGs) were identified in the *ssccp1*Δ*-1* mutant compared to the wild type. Gene ontology (GO) enrichment of the DEGs was represented in Fig. 3A, and we noticed that “oxidoreductase activity” is one of the most significant descriptors, supporting the critical role of SsCcp1 in oxidative stress response. We speculated that loss of SsCcp1 might disrupt intracellular ROS homeostasis. Hence, we visualized intracellular ROS responses using 3,3′-diaminobenzidine (DAB) staining. Indeed, *ssccp1*Δ*-1* mutant cells were stained a darker brown by the DAB dye than that of *ssccp1*Δ/*CCP1-1* strains and wild-type *MAT-1* (Fig. 3B), indicating that loss of SsCcp1 results in the accumulation of ROS. We further quantified intracellular H_2_O_2_ levels using the Amplex Red Hydrogen Peroxide Assay Kit. The results showed that H_2_O_2_ was significantly elevated in *ssccp1*Δ*-1* mutants (Fig. 3C). In short, these results suggest that deletion Ss*CCP1* led to imbalance of intracellular ROS homeostasis.

### SsCcp1 is required to maintain intracellular ROS homeostasis to regulate *S. scitamineum* mating/filamentation

**Fig 4:**
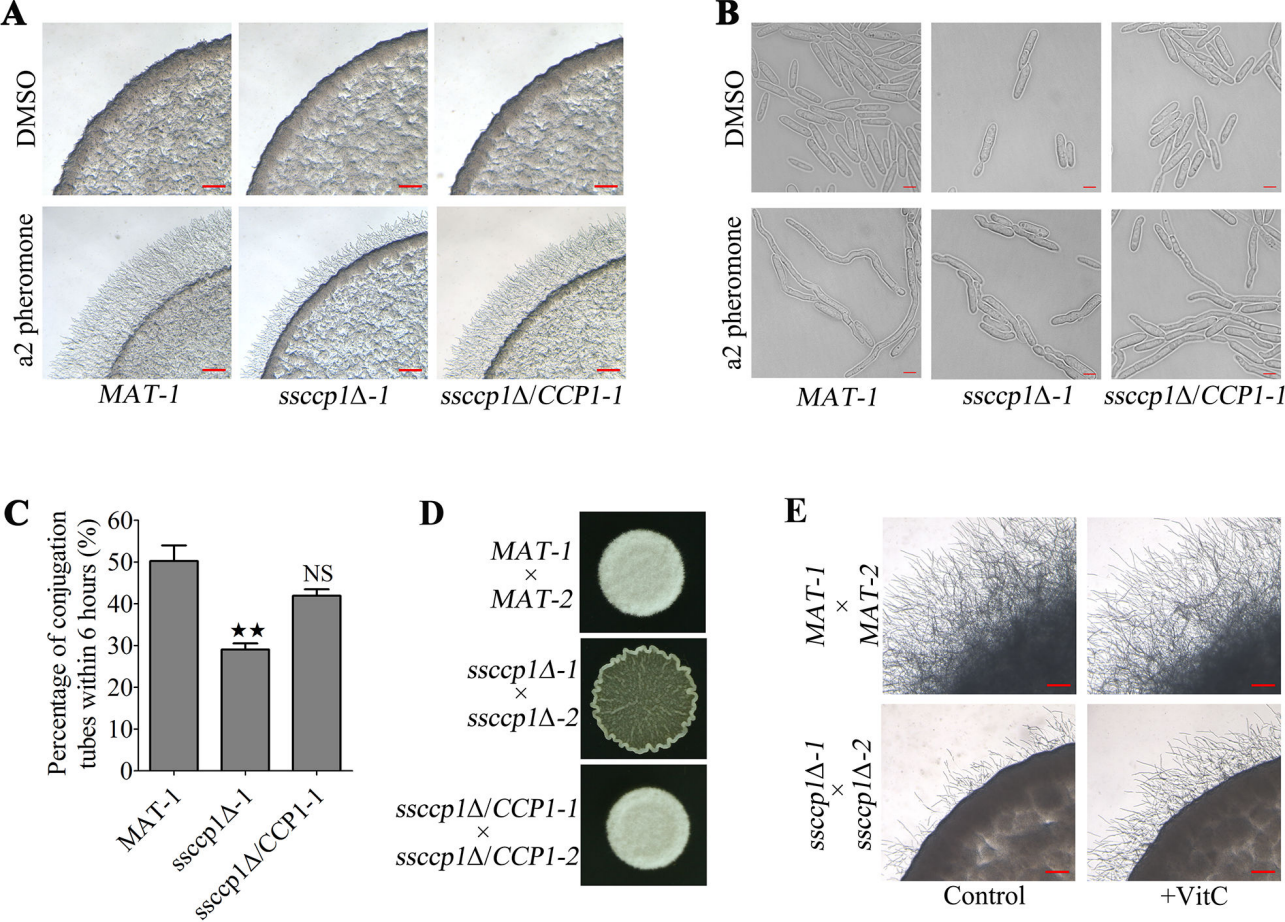
SsCcp1 maintaining ROS homeostasis is required to pheromone signal response. (**A**) The fresh haploid sporidia of the strains indicated in the below were spotted onto MM with a2 pheromone (1.0 µg/mL) for 24 h, using dimethyl sulfoxide (DMSO) as control. Images were taken at the same magnification. Scale bar is 1.0 mM. Three independent biological repeats with two replicates were performed, and representative images were displayed. (**B**) The fresh haploid sporidia of the strains indicated in the below were stimulated with a2 pheromone for 6 h and treated with DMSO for the same period of time. Images were taken at the same magnification. Scale bar is 10 µm. Three independent biological repeats with two replicates were performed, and representative images were displayed. (**C**) Bar chart depicting quantification of conjugation tube formation. Statistical significance was calculated by ANOVA, followed by Tukey’s multiple-comparison test. Error bars represent the SDs. Bar chart depicts the statistical difference among the mean values (^★★^ *P* < 0.01). NS denotes not statistically significant difference. Three independent experiments were performed. (**D**) Mating assay of wild-type, Ss*CCP1* deletion mutant, and Ss*CCP1* genetic complementation strains. The fresh haploid sporidia of the strains indicated in the left were allowed to grow until OD_600_ of 1.0 and then mixed with an equal volume of the compatible strain and spotted onto YePSA medium to incubate at 28°C. Images were taken 48 h after cultivation. The appearance of the white fuzzy colony surface indicates the formation of dikaryotic hyphae. Three independent biological repeats with two replicates were performed, and representative images were displayed. (**E**) Mating/filamentation between wild-type and Ss*CCP1* deletion mutants under exogenous vitamin C. The fresh haploid sporidia of the strains indicated in the left were allowed to grow until OD_600_ of 1.0 and then mixed with an equal volume of the compatible strain and spotted onto MM plates. Vitamin C was mixed in the MM to reach the final concentration of 1.0 mM. Images were taken 30 h after cultivation. Three independent biological repeats with two replicates were performed, and representative images were displayed.

Our previous studies have shown that ROS plays an important role in the sexual coordination of *S. scitamineum* (13). Interestingly, we found that the *SsCCP1* gene was induced during the mating/filamentation of *S. scitamineum* (Fig. S2). To evaluate the involvement of SsCcp1 in the mating/filamentation, we tested the ability of *MAT-1*, *ssccp1*Δ*-1*, and *ssccp1*Δ/*CCP1-1* strains to form conjugation hyphae upon stimulation with synthetic a2 pheromone (9). Results showed that the pseudomycelium length of the *ssccp1*Δ*-1* mutant on the YePSA plates with a2 pheromone was reduced from that of the wild-type *MAT-1* and complementation strain *ssccp1*Δ/*CCP1-1* (Fig. 4A). In response to pheromone stimulation, wild-type *MAT-1* cells underwent conjugation hyphae formation reaction, which was also observed in *ssccp1*Δ/*CCP1-1* complementation strain cells (Fig. 4B). In contrast, pheromone-stimulated *ssccp1*Δ*-1* mutant cells were weakened to form conjugation hyphae (Fig. 4B). On these grounds, counting the number of conjugation hyphae formed in haploid cells after pheromone stimulation showed a significantly lower proportion in *ssccp1*Δ*-1* mutant compared to wild-type *MAT-1* and *ssccp1*Δ/*CCP1-1* strains (Fig. 4C). Additionally, the mating/filamentation of Ss*CCP1* deletion mutants was further analyzed on YePSA medium. As expected, the wild-type and the complemented strains successfully fused and formed dikaryotic hyphae after mixing compatible strains for 48 h by the appearance of white, fuzzy colonies. However, *ssccp1*Δ*-1* × *ssccp1*Δ*-2* combination showed significant reduction in filament formation, compared with *MAT-1* × *MAT-2* combination (Fig. 4D). Based on the ROS imbalance in the cells of Ss*CCP1* deletion mutants, we tested the mating/filamentation of Ss*CCP1* deletion mutants in the presence of exogenous antioxidants. The results showed that exogenous vitamin C or vitamin E could partially restore the mating/filamentation of Ss*CCP1* deletion mutants (Fig. 4E; Fig. S3). Overall, SsCcp1 is required to maintain ROS homeostasis to regulate the mating/filamentation of *S. scitamineum*.

### SsCcp1 mediates the expression of pheromone response factor SsPrf1 essential for *S. scitamineum* mating/filamentation by maintaining ROS homeostasis

**Fig 5:**
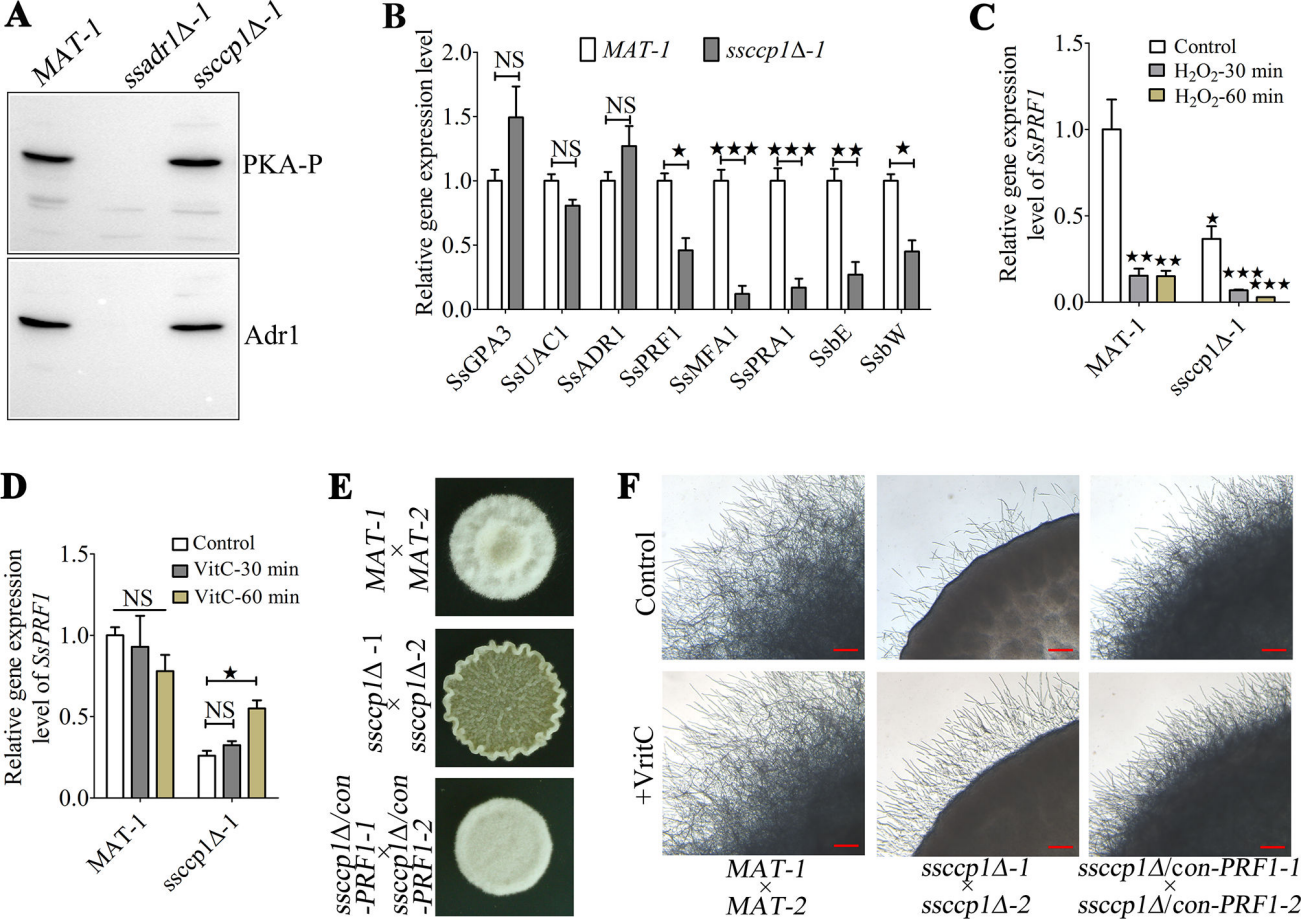
SsCcp1 is essential for pheromone-responsive gene expression. (**A**) The phosphorylation levels of PKA in the *MAT-1*, *ssadr1*Δ*-1*, and *ssccp1*Δ*-1* strains. Total protein was extracted from the fresh haploid sporidia under YePSA medium. The phosphorylated PKA was detected with the primary antibody phospho-PKA C, and the total level of SsAdr1 was determined with anti-Adr1 antibody by Western blot analysis. Three independent biological repeats were performed, and representative images were displayed. (**B**) qRT-PCR analysis of cAMP/PKA pathway and pheromone-responsive related genes in the *MAT-1* and *ssccp1*Δ*-1*. The fresh haploid sporidia of the strains were allowed to grow on MM plates for 24 h, and then, total RNA was extracted for analysis by qRT-PCR. Relative gene expression level was calculated using the −ΔΔCt method with the *ACTIN* gene as an internal control. Statistical significance was calculated by ANOVA, followed by Tukey’s multiple-comparison test. Error bars represent the SEM. Bar chart depicts the statistical difference among the mean values (^★^ *P* < 0.05, ^★★^ *P* < 0.01, ^★★★^ *P* < 0.001, ^★★★^ *P* < 0.001). NS denotes not statistically significant difference. Three independent biological repeats were performed with three technical duplicates each. (**C** and **D**) Transcriptional profile of *SsPRF1* gene in the *MAT-1* or *ssccp1*Δ*-1* mutant, with or without addition of H_2_O_2_ (1.8 mM) or vitamin C (1.0 mM). The fresh haploid sporidia of the strains were allowed to grow on liquid minimal medium treatment with H_2_O_2_ or vitamin C for 0, 30, and 60 min, and then, total RNA was extracted for analysis by qRT-PCR. Relative gene expression level was calculated using the −ΔΔCt method with the *ACTIN* gene as an internal control. Statistical significance was calculated by ANOVA, followed by Tukey’s multiple-comparison test. Error bars represent the SEM. Bar chart depicts the statistical difference among the mean values (^★^ *P* < 0.05, ^★★^ *P* < 0.01). NS denotes not statistically significant difference. Three independent biological repeats were performed with three technical duplicates each. (**E**) Mating assay of wild-type, Ss*CCP1* deletion mutant, and constitutive expression strains of *SsPRF1* in Ss*CCP1* deletion mutants. The fresh haploid sporidia of the strains indicated in the left were allowed to grow until OD_600_ of 1.0 and then mixed with an equal volume of the compatible strain and spotted onto YePSA medium to incubate at 28°C. Images were taken 48 h after cultivation. The appearance of the white fuzzy colony surface indicates the formation of dikaryotic hyphae. Three independent biological repeats with two replicates were performed, and representative images were displayed. (**F**) Mating/filamentation of wild-type, Ss*CCP1* deletion mutant, and constitutive expression strains of *SsPRF1* in Ss*CCP1* deletion mutants under exogenous vitamin C. The fresh haploid sporidia of the strains indicated in the below were allowed to grow until OD_600_ of 1.0 and then mixed with an equal volume of the compatible strain and spotted onto MM plates. Vitamin C was mixed in the MM to reach the final concentration of 1.0 mM. Images were taken 30 h after cultivation. Three independent biological repeats with two replicates were performed, and representative images were displayed.

Previously, we found that cAMP/PKA pathway (including the Gα protein SsGpa3, adenylyl cyclase SsUac1, and PKA catalytic subunit SsAdr1) regulates redox signaling essential for *S. scitamineum* mating/filamentation (13). Therefore, we assessed PKA phosphorylation levels in the *MAT-1*, *ssadr1*Δ*-1*, and *ssccp1*Δ*-1* sporidia. Unexpectedly, the phosphorylation level of PKA in *ssccp1*Δ*-1* mutants was comparable to that of wild type (Fig. 5A). Additionally, transcriptional analysis also indicated unaffected expression levels of the *SsGPA3*, *SsUAC1*, and *SsADR1* genes in wild-type and *ssccp1*Δ*-1* strains (Fig. 5B). Nevertheless, the transcription level of the pheromone response factor *SsPRF1* downstream of the cAMP/PKA pathway and its regulation of mating type genes *a* (*SsMFA1* and *SsPRA1*) and *b* (*SsbE* and *SsbW*) were significantly (*P* < 0.05) reduced in *ssccp1*Δ*-1* mutants (Fig. 5B), indicating that SsCcp1 affects the transcription of *SsPRF1*. We speculated that the down-regulation of *SsPRF1* might be due to the imbalance of ROS homeostasis in *ssccp1*Δ*-1* mutants. Therefore, we tested the transcription level of *SsPRF1* in the *MAT-1* and *ssccp1*Δ*-1* cells treated with H_2_O_2_ or antioxidant (vitamin C). The results showed that the *SsPRF1* gene in wild-type *MAT-1* and *ssccp1*Δ*-1* mutant was significantly down-regulated under the condition of exogenous H_2_O_2_ (Fig. 5C). Furthermore, exogenous vitamin C supplementation did not significantly affect the transcription of *SsPRF1* gene in wild-type *MAT-1* (Fig. 5D). However, *SsPRF1* gene in the *ssccp1*Δ*-1* mutant was partially restored by adding exogenous vitamin C for 60 min (Fig. 5D). To confirm the assertion that the decreased mating/filamentation of *SsCCP1* deletion mutants is due to the insufficient expression of *SsPRF1*, we constructed a constitutive expression strain of *SsPRF1* using the *GPA* promoter in the *ssccp1*Δ*-1* and *ssccp1*Δ*-2* background (Fig. S4). The constitutive expression of *SsPRF1* in compatible *SsCCP1* deletion strains effectively restored mating/filamentation on YePSA medium (Fig. 5E). Notably, the mating/filamentation of *SsCCP1* deletion mutants was partially restored under exogenous vitamin C, but not in *ssccp1*Δ/con*-PRF1* strains (Fig. 5F), suggesting that the transcriptional activity of *SsPRF1* activity depends on ROS homeostasis. In short, our results indicate that SsCcp1 mediates the transcriptional activity of *SsPRF1* by maintaining ROS homeostasis, thus regulating the mating/filamentous of *S. scitamineum*.

### SsCcp1 is indispensable for ROS detoxification and full pathogenicity *in planta*

**Fig 6:**
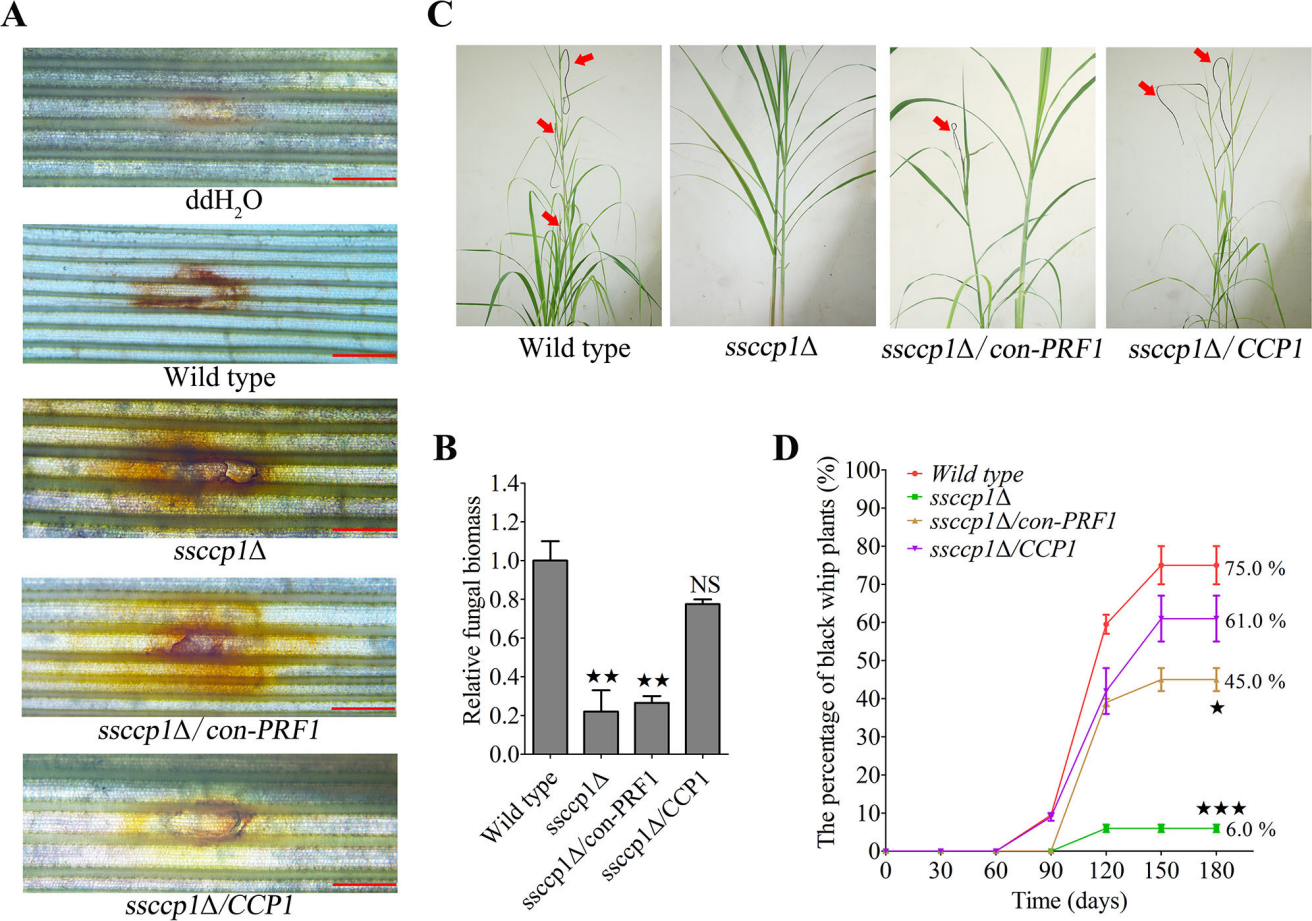
SsCcp1 is essential for ROS detoxification and full pathogenicity in *S. scitamineum*. (**A**) Visualization of host ROS response by DAB staining. The fresh of wild type (mixture of *MAT-1* ×*MAT-2*), *ssccp1*Δ (mixture of *ssccp1*Δ*-1* × *ssccp1*Δ*-2*), *ssccp1*Δ/*con-PRF1* (mixture of *ssccp1*Δ/*con-PRF1-1* × *ssccp1*Δ/*con-PRF1-2*), and *ssccp1*Δ/*CCP1* (mixture of *ssccp1*Δ/*CCP1-1* × *ssccp2*Δ/*CCP1-1*) indicated in the below were inoculated into susceptible sugarcane cultivar ROC22 by injection, using sterilized ddH_2_O as control. The inoculated sites were stained with DAB dye at 36 h post inoculation and taken pictures under the microscope. Scale bar is 5.0 mm. Three independent biological repeats with three replicates were performed, and representative images were displayed. (**B**) qRT-PCR analysis of relative fungal biomass. The fresh haploid sporidia of the strains indicated in the (A) were inoculated into susceptible sugarcane cultivar ROC22 by injection, and then, total DNA was extracted from infected sugarcane stems after 3 days post infection. The fungal *ACTIN* gene was used for estimation of relative fungal biomass, and the plant *GADPH* gene, as an internal control. Statistical significance was calculated by ANOVA, followed by Tukey’s multiple-comparison test. Error bars represent the SEM. Bar chart depicts the statistical difference among the mean values (^★★^ *P* < 0.01). NS denotes not statistically significant difference. Three independent biological repeats were performed with three technical duplicates each. (**C** and **D**) For plant infection assay, the fresh haploid sporidia of the wild type (mixture of *MAT-1* × *MAT-2*), *ssccp1*Δ (mixture of *ssccp1*Δ*-1* × *ssccp1*Δ*-2*), *ssccp1*Δ/*con-PRF1* (mixture of *ssccp1*Δ/*con-PRF1-1* × *ssccp1*Δ/*con-PRF-2*), and *ssccp1*Δ/*CCP1* (mixture of *ssccp1*Δ/*CCP1-1* × *ssccp2*Δ/*CCP1-2*) indicated in the below were inoculated into susceptible sugarcane cultivar ROC22 of five- to six-leaf seedling stage by injection. For each combination, at least two independent infections with 15 seedlings were performed. Disease symptoms were recorded 180 days after inoculation (dpi). The symptom of “black whip” was denoted by red arrows and represents the most common infection symptom. Statistical significance was calculated by ANOVA, followed by Tukey’s multiple-comparison test. Error bars represent the SDs. Curve chart depicts the statistical difference among the mean values (^★^ *P* < 0.05, ^★★★^ *P* < 0.01).

The Ccp1 protein is capable of reducing H_2_O_2_ to water in fungal pathogen (32). Thus, we investigated the role of SsCcp1 in ROS detoxification by the DAB staining assay. The results showed that at the early stage of infection, the accumulation of H_2_O_2_ in the stem of a sugarcane seedling inoculated with the *ssccp1*Δ (mixture of *ssccp1*Δ*-1* × *ssccp1*Δ*-2*) and *ssccp1*Δ/*con-PRF1* (mixture of *ssccp1*Δ/*con-PRF1-1* × *ssccp1*Δ/*con-PRF1-1*) mutants exhibited a larger zone than those infected with the wild-type (mixture of *MAT-1* × *MAT-2*) and *ssccp1*Δ/*CCP1* (mixture of *ssccp1*Δ/*CCP1−1* × *ssccp2*Δ/*CCP1-2*) strains (Fig. 6A). Consistently, the relative fungal biomass was significantly reduced at 3 days post infection (dpi) with *ssccp1*Δ and *ssccp1*Δ/*con-PRF1* mutants, compared to the wild-type and genetic complementary strain combinations (Fig. 6B), suggesting that SsCcp1 is instrumental in ROS detoxification.

Given that SsCcp1 is important for *S. scitamineum* mating/filamentous growth and ROS detoxification, we speculate that it may be involved in pathogenicity. Hence, we inoculated the *ssccp1*Δ (mixture of *ssccp1*Δ*-1* × *ssccp1*Δ*-2*), *ssccp1*Δ/*con-PRF1* (mixture of *ssccp1*Δ/*con-PRF1-1* × *ssccp1*Δ/*con-PRF1-2*), and *ssccp1*Δ/*CCP1* (mixture of *ssccp1*Δ/*CCP1-1* × *ssccp2*Δ/*CCP1-2*) sporidia into a susceptible sugarcane cultivar ROC22 by injection. Inoculation with the wild-type *MAT-1* × *MAT-2* combination served as positive control. Such inoculated seedlings were allowed to grow at the greenhouse for approximately 180 days until the typical disease “black whip” symptom was observed. Our results showed that the wild type led to 75% of the infected seedlings with black whip symptoms, while the *ssccp1*Δ/*CCP1* strain caused approximately 61% (Fig. 6C and D). In contrast, typical whip symptom in the *ssccp1*Δ strain-infected seedlings displayed significantly reduced pathogenicity, with only around 6.0% (Fig. 6C and D). Although 45% of sugarcane seedlings induced by *ssccp1*Δ/*con-PRF1* mutant to form black whip were lower than those of the wild type, infection was significantly higher than for the infection with the *ssccp1*Δ mutant (Fig. 6C and D). Taken together, these findings indicate that SsCcp1 is required for ROS detoxification and full pathogenicity of *S. scitamineum*.

## DISCUSSION

Plants’ growth and development in nature are affected by exposure to various pathogens. ROS are one of the most powerful weapons for plants to resist pathogens in the early stage of infection (34, 35). Mitochondria are the main source of ROS in cells (36, 37). In eukaryotes, cytochrome *c*-peroxidase is nuclearly encoded and present in the mitochondrial intermembrane space, where it catalyzes the reduction of H_2_O_2_ to water by ferrocytochrome (21, 23, 31). In this study, we characterized the gene encoding cytochrome *c*-peroxidase in *S. scitamineum* and found that the transcription levels of *SsCCP1* were significantly induced during the mating/filamentation and the transcriptional expression of SsCcp1 was remarkably increased by H_2_O_2_ treatment. Furthermore, the *SsCCP1* deletion mutants hardly grew under H_2_O_2_ stress conditions and observably weakened in removing plant-produced H_2_O_2_, indicating that SsCcp1 is essential for ROS detoxification. This is also supported by the evidence that Ccp1 contributes to ROS detoxification and protects cells from the effects of H_2_O_2_ (30, 32). Nevertheless, SsCcp1 is involved in oxidative stress resistance independently of Hog1-MAPK pathway. Therefore, the regulatory pathway of SsCcp1 further needs to be explored.

ROS homeostasis plays an important role in the normal physiological activities of organisms (37), as high ROS levels have toxicity effects, while applicable levels of ROS positively regulate signaling (38, 39). Here, we found that the accumulation of H_2_O_2_ was significantly increased in the *SsCCP1* deletion mutant, indicating that SsCcp1 plays a key role in maintaining ROS homeostasis. This is consistent with the result that Ccp1 activity contributes to ROS homeostasis in *C. albicans* and *S. cerevisiae* (22, 29).

Cytochrome *c*-peroxidase is well conserved in fungi (30). We found that *SsCCP1* deletion mutants were significantly reduced in conjugation tube formation and mating/filamentation, indicating that SsCcp1 is an essential regulator of the sexual reproduction. In contrast, Ccp1 is a negative regulator of hyphal growth in human pathogen *C. albicans* (29). However, there have been no reports indicating that Ccp1 regulates the sexual reproduction in phytopathogenic fungi. We were interested to find that *SsPRF1* was significantly down-regulated in the *SsCCP1* deletion mutant or under highly concentrated H_2_O_2_ conditions, and the restoration of *SsPRF1* transcription by exogenous vitamin C in *ssccp1*Δ*-1* mutants further supports the relationship between ROS homeostasis and *SsPRF1* expression. Additionally, exogenous vitamin C or vitamin E could partially restore the mating/filamentation of Ss*CCP1* deletion mutants, and the constitutive expression of *SsPRF1* basically restored the mating/filamentation of Ss*CCP1* deletion mutants, suggesting that SsCcp1 mediates the expression of SsPrf1 by maintaining intracellular ROS homeostasis to regulate the mating/filamentation of *S. scitamineum*. Nevertheless, our study raised a question that needed to be explored in the future: how does the ROS homeostasis maintained by SsCcp1 regulate SsPrf1. For example, the activity of direct regulator of the transcription factor Prf1 was analyzed under oxidation-reduction conditions. In the maize pathogenic fungus *U. maydis*, several regulators of the transcription factor Prf1 have been investigated, such as Rop1, Pac2, Hap2, and Med1 (40 – 44). On the other hand, we found that the pathogenicity of *SsCCP1* deletion mutants was significantly reduced. This is in accord with what has been reported that inactivation of Ccp1 decreased the pathogenicity of *Magnaporthe oryzae*, *C. albicans*, and *Botrytis cinerea* (29, 32, 45), but not in *Cryptococcus neoformans* (30).

In conclusion, our study demonstrated that SsCcp1 is essential for ROS detoxification, and its plays an important role in mediating the SsPrf1 by maintaining ROS homeostasis, thereby regulating the mating/filamentation of *S. scitamineum*. These results provide a theoretical basis for developing new strategies to control the disease.

## MATERIALS AND METHODS

### Strains and growth conditions

The *Escherichia coli* DH5α strain was employed for cloning purposes and amplification of plasmid DNA. The wild-type *MAT-1* (*a1 b1*) and *MAT-2* (*a2 b2*) were isolated and maintained in our laboratory. Details of all fungal strains used in this study can be found in Table S1 in the supplemental material, respectively. Cultures of *S. scitamineum* cells were cultivated at 28°C in yeast extraction-peptone-sucrose (YePS) (pH 6.5) liquid medium (8) or minimal medium (MM) (46), containing 100 µg/mL ampicillin. For mating/filamentous and/or oxidative stress assay, fungal strains were spotted onto YePS medium or minimal medium supplemented with 2% agar and then incubated at 28°C.

### Plasmid and strain construction

To generate *SsCCP1* compensated plasmid, a 1-kb segment of the *SsCCP1* open reading frame and 0.3 kb downstream of the *SsCCP1* stop codon were PCR amplified from genomic DNA. The resulting PCR product was integrated into the pEASY-COM vector (constructed by our laboratory) (14) using ClonExpress II One Step Cloning Kit (Vazyme), generating plasmid pEASY-COM-*SsCCP1*. To generate *SsCCP1* overexpression plasmid, the *SsCCP1* gene was PCR amplified from cDNA and then integrated into the pEASY-OE vector (constructed by our laboratory) (14) by ClonExpress II One Step Cloning Kit (Vazyme), resulting in plasmid pEASY-OE-*SsCCP1*. For the promoter GPA-*SsPRF1* fusion, the *SsPRF1* gene was PCR amplified from cDNA and then integrated into the pEASY-con vector (constructed by our laboratory) by ClonExpress II One Step Cloning Kit (Vazyme), resulting in plasmid pEASY-con-*SsPRF1*. Refer to Table S2 for the primer details.

All mutants were constructed by polyethylene glycol (PEG)-mediated protoplast transformation using two truncated resistance marker fragments, as described previously (13, 47). To generate deletion mutants, 1.5-kb flanking regions upstream (or downstream) of target gene were fused with the upstream (downstream) of the truncated hygromycin resistance (*HYG^R^*) gene, respectively. The resident target gene was replaced with the *HYG^R^* gene by homologous recombination, individually. The deletion mutants were selected with 200 µg/mL hygromycin B (Merck). Correct integration was verified by PCR amplification, Southern blot, and qRT-PCR assays. For complementation of *SsCCP1* gene, two truncated and partially overlapped zeocin resistance (*ZEO^R^*) gene fragments, including the fusion of *SsCCP1* gene and its native promoter, were PCR amplified from plasmid pEASY-COM-*SsCCP1*. The resulting PCR products were mixed and transformed into *ssccp1*Δ*-1* and *ssccp1*Δ*-2* mutant by PEG-mediated protoplast transformation, respectively. The *HYG^R^* gene in the *ssccp1*Δ*-1* or *ssccp1*Δ*-2* mutant was replaced with *SsCCP1* and *ZEO^R^* gene. The genetic complementation strains were selected with 100 µg/mL Zeocin (Invitrogen). For the constitutive expression of *SsPRF1*, two truncated and partially overlapped *ZEO^R^* gene fragments containing the *GPA* promoter fused with the *SsPRF1* gene were PCR amplified from plasmid pEASY-con-*SsPRF1*. The resulting PCR products were mixed and transformed into *ssccp1*Δ*-1* or *ssccp1*Δ*-2* mutant by PEG-mediated protoplast transformation, respectively. The transformants were selected with 100 µg/mL Zeocin (Invitrogen). For the overexpression of *SsCCP1*, a nonfunctional DNA region in the *sshog1*Δ strains was replaced by two truncated and partially overlapped *ZEO^R^* gene fragments containing the *GPA* promoter fused with the *SsCCP1* gene. Two truncated and partially overlapped DNA fragments were PCR amplified from plasmid pEASY-con-*SsPRF1*. The transformants were selected with 100 µg/mL Zeocin (Invitrogen). Correct integration was verified by PCR amplification and qRT-PCR assays. The primers used in this study are listed in Table S2.

### DNA and RNA procedures

DNA isolation from *S. scitamineum* was performed using SDS-based DNA extraction methods described in references (8, 48). For Southern blot assay, genomic DNA of wild type and derivatives were digested with the restriction enzyme *Hind* III and *BamH* I at 37°C overnight. The pDAN vector served as positive control, and the *HPT^R^* sequence served as the probe. Probed bands larger than 3.0 kb in size in the deletion mutants confirmed the correct gene replacement events. Total RNA was extracted from fresh cells using RNeasy Mini Kit (Qiagen) according to the manufacturer’s instructions. The HiScript II 1st Strand cDNA Synthesis Kit (Vazyme) was used to remove residual DNA from RNA samples and synthesize cDNA.

### Transcriptome analysis and quantitative RT-PCR

For transcriptome analysis, the fresh haploid sporidia of *MAT-1* and *ssccp1*Δ*-1* strains allowed to grow in YePSA medium at 28°C for 24 h , and then, total RNA was extracted and sent to Novogene Technology Co., Ltd., for transcriptome analysis. GO enrichment analysis of DEGs with *P*-value <0.05 (6). qRT-PCR analyses were performed as previously described (49). qRT-PCR experiments were performed with ChamQ SYBR qPCR Master Mix (Vazyme) on the QuantStudio 6 Flex (Life Technologies). The relative gene expression level was calculated using the −ΔΔCt method (50), with the cytoskeletal protein gene *ACTIN* as an internal control. Three independent biological replicates, and two technical replicates were performed.

### Western blotting

*S. scitamineum* cells were harvested and washed once with 1× phosphate-buffered saline and then rapidly ground in liquid nitrogen, as described previously (49). The debris was resuspended in lysis buffer [50 mM Tris–HCl (pH 7.9), 50 mM NaCl, 0.5 mM EDTA, 5% glycerin] supplemented with 1× protease inhibitor mixture (Beyotime) and/or 1× phosphatase inhibitor (Beyotime). The resulting suspension was cleared by centrifugation at 15,000 rpm for 2 min at 4°C. The supernatant was collected and supplemented with 5× protein loading buffer (Sangon Biotech) and then boiled for 5 min at 98°C. The dissolved proteins were subjected to 10% SDS-PAGE. For phosphorylation assays, phosphorylation of SsHog1 was detected by the primary antibody phospho-p38 MAPK (Thr180/Tyr182) (Cell Signaling Technology), and phosphorylated PKA was determined by the primary antibody phospho-PKA C (Cell Signaling Technology). Commercially available anti-tubulin and prepared antibodies (anti-Hog1, anti-Adr1, and anti-Ccp1 were prepared from Genecreate Biological Engineering Company) were used to detect respective proteins. Horseradish peroxidase-conjugated anti-mouse (HUABIO) or anti-rabbit IgG (Sigma) was used as secondary antibodies. Blot signals were displayed by using the enhanced chemiluminescence (Bio-Rad) method.

### Pheromone stimulation and H_2_O_2_ treatment

To test pheromone stimulation, the fresh haploid sporidia of the strains allowed to grow in YePS liquid medium until OD_600_ of 1.0 at 28°C and then spotted onto MM plates with a2 pheromone for 24 h or harvested cells for microscopic observations after 6 h of incubation at 28°C. Synthetic a2 pheromone dissolved in dimethyl sulfoxide (DMSO) was added to a final concentration of 1.0 µg/mL. DMSO was added as a negative control. Three independent biological repeats with two replicates were performed.

For H_2_O_2_-induced gene expression and protein extraction, overnight cultures of *S. scitamineum* haploid were grown in liquid minimal medium, adjusted to an OD_600_ of 1.0 in liquid minimal medium treatment with or without 1.8 mM H_2_O_2_, and further incubated for 30 or 60 min at 28°C. Cells were collected by centrifugation and flash frozen in liquid nitrogen for subsequent total RNA and protein isolation.

### DAB stain and quantification of H_2_O_2_

Intracellular H_2_O_2_ of *S. scitamineum* cells was detected by DAB (Sigma) staining, which is oxidized by H_2_O_2_ to give a brown color (13). The fresh haploid sporidia of the strains were allowed to grow until OD_600_ of 1.0 at 28°C in a shaking incubator and then stained with DAB dye for 5 min. Photographs were captured using the Leica DMI8 Inverted Fluorescence Microscope. For visualization of host ROS response, the fresh haploid strains were cultured in YePS liquid medium at 28°C overnight and adjusted to an OD_600_ of 1.0 with sterilized ddH_2_O. Haploid strains were mixed 1:1 with the opposite mating types, respectively, and then inoculated into susceptible sugarcane cultivar ROC22 by injection, using sterilized ddH_2_O as control. The inoculated sites were stained with DAB (pH 3.8) dye at 36 h post inoculation and then destained with clearing solution (ethanol:acetic acid, 94:4, vol/vol) for 1 h. Three independent biological repeats with three replicates were performed.

To quantify for intracellular H_2_O_2_ of *MAT-1* and its derivatives, overnight cultures of haploid strains were grown in YePS liquid medium, adjusted an OD_600_ of 1.0 with sterilized ddH_2_O, and smeared on MM plates and further incubated for 24 h at 28°C. Cells were collected to measure the H_2_O_2_ concentration using the Amplex Red Hydrogen Peroxide/Peroxidase Assay Kit (Thermo Fisher) according to the manufacturer’s instructions. Three independent experiments were performed in triplicate.

### Mating/filamentous growth assay

Mating/filamentous assays were performed as described in reference (14). The fresh haploid strains were cultured in YePS liquid medium at 28°C overnight and adjusted to an OD_600_ of 1.0 with sterilized ddH_2_O. Haploid strains were mixed 1:1 with the opposite mating types, respectively, and then spotted onto YePS medium supplemented with 2% agar (or spotted onto MM plates with or without exogenous vitamin C or vitamin E). The plates were then incubated at 28°C for 12–48 h. Three independent biological repeats with two replicates were performed.

### Oxidative stress sensitivity spot assay

The fresh haploid sporidia of the strains were allowed to grow until OD_600_ of 1.0 at 28°C, serially diluted 10-fold three times (final dilution, 1:10^3^), and spotted onto YePS plates with or without 1.8 mM H_2_O_2_. Qualitative growth assessment was carried out by photographing the plates 3 to 4 days later. Three independent biological repeats with two replicates were performed.

### Fungal biomass assessment and pathogenicity assay

Fungal biomass assays were performed as described before (9). The fresh haploid strains were cultured in YePS liquid medium at 28°C overnight, adjusted to 1 × 10^6^ cells/mL with sterilized ddH_2_O, and further mixed 1:1 with a compatible mating partner. A total of 0.5 mL of the resulting cell suspension was used to inoculate the susceptible sugarcane cultivar ROC22 at five-/six-leaf stage. Total DNA was isolated from inoculated sugarcane tissue after 3 days post infection. qRT-PCR analysis of relative fungal biomass was performed using the fungal *ACTIN* gene as a reference, and the sugarcane glyceraldehyde dehydrogenase (*GAPDH*) gene served as an internal control. Three independent biological repeats were performed with three technical duplicates each.

For the pathogenicity assay, the fresh haploid strains were grown overnight at 28°C in YePS liquid medium and then adjusted to an OD_600_ of 1.0 with sterilized ddH_2_O and further mixed 1:1 with the opposite mating types, respectively. Next, a total of 0.5 mL of the resulting cell suspension was used to inoculate five-/six-leaf stage of the susceptible sugarcane cultivar ROC22 by injection. Inoculated plants were grown in a greenhouse with a natural cycle of day and night for 180 days. Two biological repeats were used for plant infection experiments, and each replicate involved the infection of at least 15 plants. Disease symptoms were photographed and recorded at post-inoculation. The symptoms of “black whip” plants/total seedlings were indicated.

### Statistical analysis

Statistical significance was determined using by one-way analysis of variance, followed by Tukey’s multiple-comparison test. Error bars represent standard deviations or standard error of mean. Results were considered significant at *P*-value <0.05. Bar chart and curve chart were generated using GraphPad Prism 5 software.

## Data Availability

RNA-seq data for this study have been deposited in the GEO database (GSE240666). Additional data can be found in the supplemental material files.

## SUPPLEMENTAL MATERIAL

Supplemental material spectrum.02057-23-s0001.pdf Fig. S1 to S4; Tables S1 and S2.

ASM does not own the copyrights to Supplemental Material that may be linked to, or accessed through, an article. The authors have granted ASM a non-exclusive, world-wide license to publish the Supplemental Material files. Please contact the corresponding author directly for reuse.
