# Supplementary material for: Cytochrome c-peroxidase modulates ROS homeostasis to regulate the sexual mating of Sporisorium scitamineum
Source: Microbiol Spectr. 2023 Oct 11;11(6):e02057-23. doi: 10.1128/spectrum.02057-23 (PMC10714796; doi:10.1128/spectrum.02057-23)
Supplement: Supplemental material — Fig. S1 to S4; Tables S1 and S2. [file spectrum.02057-23-s0001.pdf]

Supporting Information

Cytochrome *c*-Peroxidase Modulates ROS Homeostasis to Regulate the Sexual Mating of *Sporisorium scitamineum*

To whom correspondence should be addressed. Email: changcq@scau.edu.cn (C Chang).

This PDF file includes

Figure and Table Legends

FIG S1 Generation and verification of mutants of *S. scitamineum*. (A) PCR amplification using the locus specific primers (listed in Table S1) to confirm the replacement of targeted genes with the *HPT* selection marker (gene deletion) or replacement of *HPT* selection marker with targeted genes (genetic complementation and overexpression). Molecular markers in bp were labeled. (B) Southern blot analysis was performed for confirming the *SsCCP1* deletion mutants. The genomic DNA of the samples (*MAT-1*, *MAT-2*, *ssccp1*Δ*-1*, and *ssccp1*Δ*-2* were digested with the restriction enzyme *Hind* III and *BamH* I at 37 °C for overnight. Fragment of *HPT* gene was used as the probe. The 5755 bp band of the *HPT* fragment served as a positive control of the experimental procedure. Probed bands of 5301 bp size in the *SsCCP1* deletion mutants confirmed the correct gene replacement events, respectively. (C) qRT-PCR analysis of *SsCCP1* gene in the *MAT-1*, *MAT-2*, *ssccp1*Δ*-1*, *ssccp1*Δ*-2*, *ssccp1*Δ*/CCP1-1*, and *ssccp1*Δ*/CCP1-1* strains. The fresh haploid sporidia of the strains indicated in the below were allowed to grow on minimal medium for 24 h, and then extracted total RNAs to analyse by qRT-PCR. Relative gene expression level was calculated with -ΔΔCt method with the *ACTIN* gene as an internal control. Statistical significance was calculated by ANOVA, followed by Tukey's multiple-comparison test. Error bars represent the standard error of mean (SEM). Bar chart depicts the statistical difference among the mean values (★★★p<0.001). NS denote not statistically significant difference. Three independent biological repeats were performed with three technical duplicates each.

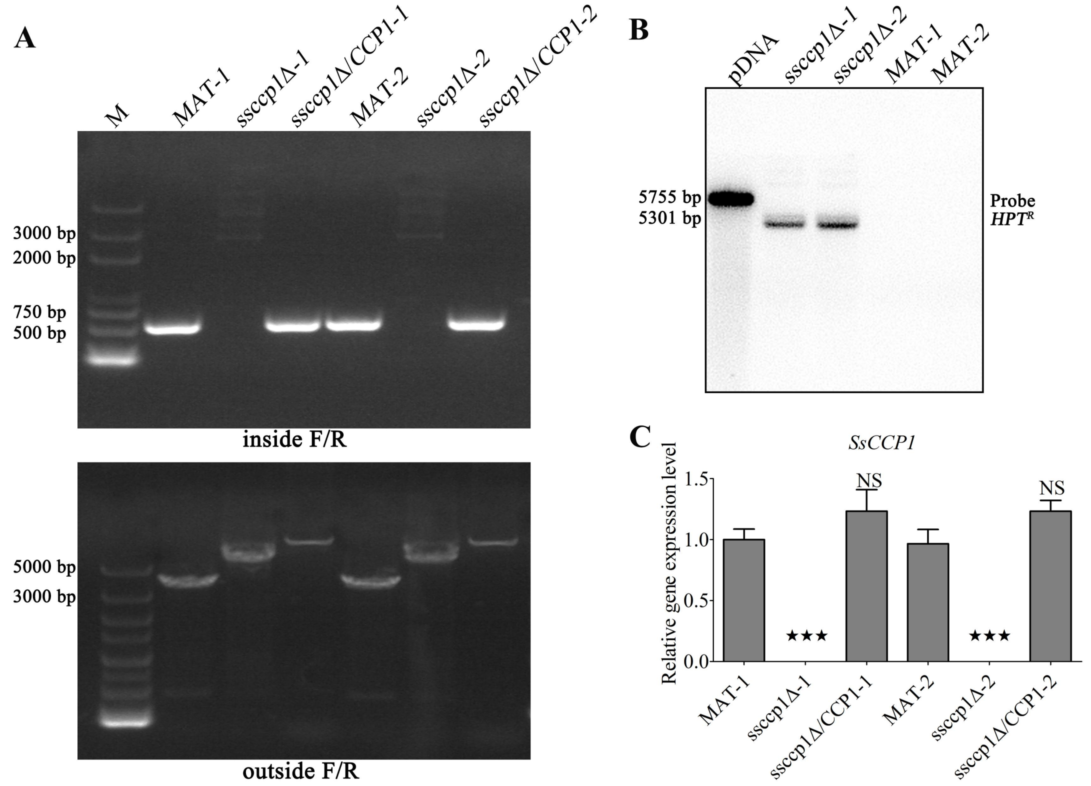
A
M
MAT-1
ssccp1Δ-1
ssccp1Δ/CCP1-1
MAT-2
ssccp1Δ-2
ssccp1Δ/CCP1-2
3000 bp
2000 bp
750 bp
500 bp
inside F/R
5000 bp
3000 bp
outside F/R
B
pDNA
ssccp1Δ-1
ssccp1Δ-2
MAT-1
MAT-2
5755 bp
5301 bp
Probe
HPTR
C
SsCCP1
Relative gene expression level
1.5
1.0
0.5
0.0
NS
NS
★★★
★★★
MAT-1
ssccp1Δ-1
ssccp1Δ/CCP1-1
MAT-2
ssccp1Δ-2
ssccp1Δ/CCP1-2

FIG S1

FIG S2 Transcriptional patterns of *SsCCP1* gene during mating/filamentation of *S. scitamineum*. qRT-PCR analysis of *SsCCP1* gene in the mating/filamentation of *S. scitamineum*. The fresh haploid sporidia of the wild type (mixture of *MAT-1*×*MAT-2*) were allowed to grow on minimal medium for 0, 12, 24, 36, 48, 60, and 72 h, respectively, and then extracted total RNAs to analyse by qRT-PCR. Statistical significance was calculated by one-way analysis of variance (ANOVA), followed by Tukey's multiple-comparison test. Error bars represent the standard error of mean (SEM). Bar chart depicts the statistical difference among the mean values (★$p<0.05$, ★★$p<0.01$). NS denote not statistically significant difference. Three independent biological repeats were performed with three technical duplicates each.

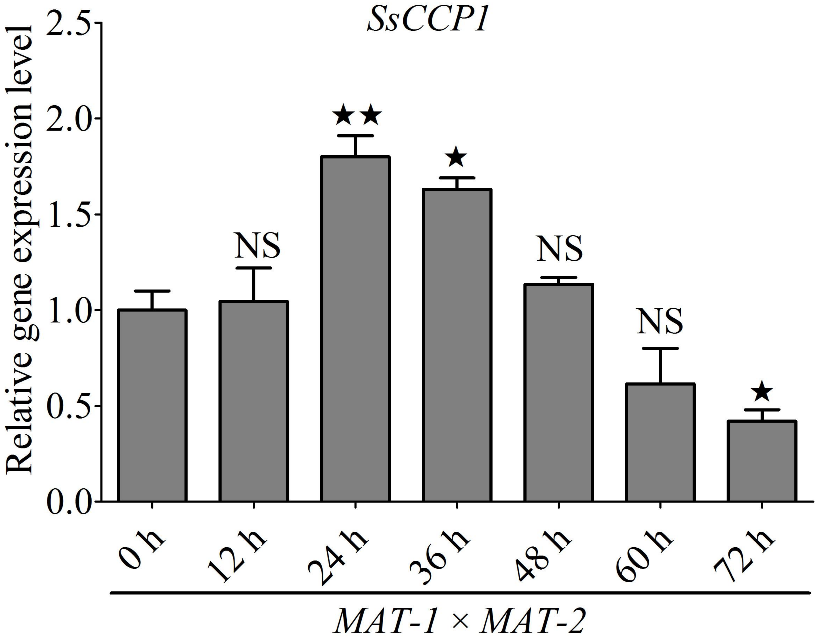

FIG S2

FIG S3 Mating/filamentation of *S. scitamineum* under exogenous vitamin E. The fresh haploid sporidia of the strains indicated in the left were allowed to grow till OD_{600} of 1.0, and then mixed with an equal volume of the compatible strain and spotted onto MM plates. Vitamin E was mixed in the MM to reach the final concentration of 1.0 mM. Images were taken 30 h after cultivation. Three independent biological repeats with two replicates were performed, and representative images were displayed.

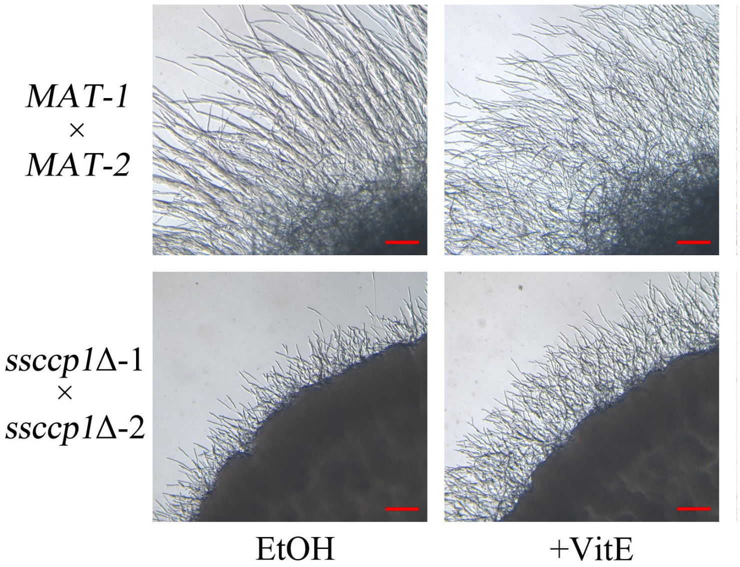

FIG S3

FIG S4 qRT-PCR analysis of *SsPRF1* gene in the genetic constitutively strains. qRT-PCR analysis of *SsPRF1* gene in the *MAT-1*, *MAT-2*, *ssccp1*Δ/*con-PRF1-1*, and *ssccp1*Δ/*con-PRF1-2* strains. The fresh haploid sporidia of the strains indicated in the below were allowed to grow on minimal medium for 24 h, and then extracted total RNAs to analyse by qRT-PCR. Relative gene expression level was calculated with -ΔΔCt method with the *ACTIN* gene as an internal control. Statistical significance was calculated by ANOVA, followed by Tukey's multiple-comparison test. Error bars represent the standard error of mean (SEM). Bar chart depicts the statistical difference among the mean values (**$p<0.01$). NS denote not statistically significant difference. Three independent experiments were performed in triplicate.

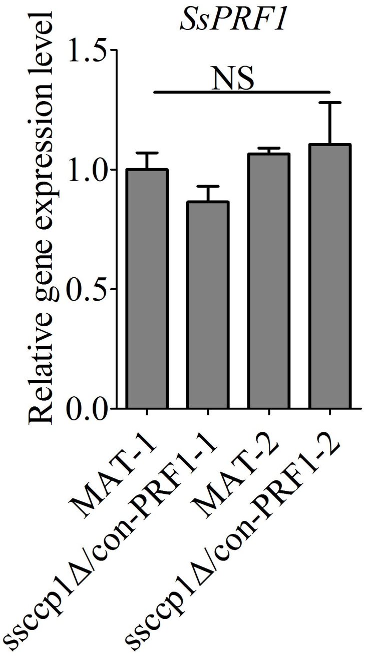

FIG S4

Table

Table S1. Details of strains generated in this study.

Strain	Relevant Genotype	Resistance marker	Source
MAT-1	*a1*, *b1*		Reference (8)
MAT-2	*a2*, *b2*		Reference (8)
ssccp1Δ-1	*a1*, *b1*; *ccp1Δ*	Hygromycin	This study
ssccp1Δ-2	*a2*, *b2*; *ccp1Δ*	Hygromycin	This study
ssccp1Δ/CCP1-1	*a1*, *b1*; *ccp1Δ*; *CCP1*	Zeocin	This study
ssccp1Δ/CCP1-2	*a2*, *b2*; *ccp1Δ*; *CCP1*	Zeocin	This study
ssccp1Δ/con-PRF1-1	*a1*, *b1*; *ccp1Δ*; *PRF1*$^{-con}$	Hygromycin, Zeocin	This study
ssccp1Δ/con-PRF1-2	*a2*, *b2*; *ccp1Δ*; *PRF1*$^{-con}$	Hygromycin, Zeocin	This study
sshog1Δ/OE-CCP1-1	*a1*, *b1*; *ccp1Δ*; *CCP1*$^{-OE}$	Hygromycin, Zeocin	This study
sshog1Δ/HOG1-1	*a1*, *b1*; *ccp1Δ*; *HOG1*	Zeocin	Reference (33)
sshog1Δ-1	*a1*, *b1*; *hog1Δ*	Hygromycin	Reference (33)

Table S2. The primers and sequences used in this study.

The primers	Sequence (5’- - 3’)
The primers for genetic deletion	
pDAN-F	GTCGTGACTGGGAAAACCCTG
LB-226-R	GGTCAAGACCAATGCGGAGC
pDAN-R	TCACACAGGAAACAGCTATGACC
RB-225-F	GCAAGACCTGCCTGAAACCG
SsCCP1-LB-F	ATCATCGTCCGTGCTGCCATC
SsCCP1-LB-R	GTCGTGACTGGGAAAACCCTGTGATGTTGCAGGAAGGAGGAAGG
SsCCP1-RB-F	GGTCATAGCTGTTTCCTGTGTGACCGGTTTCGATGGTCCCTGG
SsCCP1-RB-R	CGAAGCTGACGAGTGGAGGAA
The primers for genetic complementation	
SsCCP1-COM-F	ATCTGATCCAAGCTCAAGCTGTTCATTCCACGGTGCGAAGC
SsCCP1-COM-R	AGCAAGATCTAATCAAGCTTGACGCGCAGACTAGAACGCA
COM-LB-F	CCTCGAGCGATCCTTGAAGC
COM-LB-R	AGCGGGCAGTTCGGTTTCA
COM-RB-F	CAAGAACAAGCGCTGTCGCC
COM-RB-R	CGAGCATTCACTAGGCAACCA
The primers for genetic overexpression and constitutive expression	
SsPRF1-con-F	AACCAAAACACTCTTCCACCATGCGAGACCAAGCTACCACG
SsPRF1-con-R	AGCAAGATCTAATCAAGCTTCTACGTCGAGGCGGACTGCTG
SsCCP1-OE-F	AACCAAAACACTCTTCCACCATGGCCTCTCTTCGCACTG
SsCCP1-OE-R	AGCAAGATCTAATCAAGCTTTTACTTGGCGTTCTCCTGCTCGT
OE-LB-F	GAAAGGTGCGACGGTGTGC
OE-LB-R	GGCTGTCGAAAGGTCAGGTCT
OE-RB-F	AGTTGACCAGTGCCGTTCCG
OE-RB-R	AGCGACGAACCTTGCCATCA
The primers for gene knockout identification	
SsCCP1-inside-F	ATGGCCTCTCTTCGCACTGG

SsCCP1-inside-R	CGGGAGCGAATCGCATCGTA
SsCCP1-outside-F	ATGAGCGGCCCGAGAATCG
SsCCP1-outside-R	GTCTGGGGCTGCACATTGGA
The primers for qRT-PCR	
qRT-ACTIN-F	CAGCTCGATGAAGGTCAAGAT
qRT-ACTIN-R	CACATCTGCTGGAAGGTAGAG
qRT-SsCCP1-F	CACCAACGAGTACTTCAACCT
qRT-SsCCP1-R	GCTCTTGGTCGACTTGTCTT
qRT-SsGPA3-F	CAAGTACATTCTCTGGCGTTTC
qRT-SsGPA3-R	AGTCGGATGTTGCTCGTATC
qRT-SsUAC1-F	GCACGACAACCTGAGTGTAA
qRT-SsUAC1-R	GCCGTCAAAGAGACCAAAGA
qRT-SsADR1-F	CGTGCTGCTCTACGAAATGC
qRT-SsADR1-R	AAGATCCTTGACGCCCGTTT
qRT-SsPRF1-F	CAAGCAGTGTCACCGTTAGA
qRT-SsPRF1-R	GGAGAGCAAGGATGCAAGAT
qRT-SsMFA1-F	ATGCTTTCCATCTTTACCCAGA
qRT-SsMFA1-R	GTGCAGCTAGAGTAGCCAAG
qRT-SsPRA1-F	GGACGCTATCACCCAATCTTAC
qRT-SsPRA1-R	TCTCCAACATGGCAACACTC
qRT-SsbE-F	TGAAAGTTCTCATGCAAGCC
qRT-SsbE-R	TGAGAGGTCGATTGAGGTTG
qRT-SsbW-F	CCAGCAGTCCAATGGAGAAA
qRT-SsbW-R	AGAAAGGGTTGGACGACAAG
qRT-SsHOG1-F	AGTGGACGTACTTGAGACCT
qRT-SsHOG1-R	ATCTCGCCTCTTGAGGACAT
qRT-GADPH-F	CACGGCCACTGGAAGCA
qRT-GADPH-R	TCCTCAGGGTTCCTGATGCC
